# Association of mixed polycyclic aromatic hydrocarbons exposure with oxidative stress in Korean adults

**DOI:** 10.1038/s41598-024-58263-9

**Published:** 2024-03-29

**Authors:** Ji Young Ryu, Dong Hyun Hong

**Affiliations:** Department of Occupational and Environmental Medicine, Inje University Haeundae Paik Hospital, 875 Haeun-daero, Haeundae-gu, Busan, 48108 South Korea

**Keywords:** Environmental impact, Public health, Epidemiology

## Abstract

Polycyclic aromatic hydrocarbons (PAHs) are widespread pollutants associated with several adverse health effects and PAH-induced oxidative stress has been proposed as a potential mechanism. This study evaluated the associations of single and multiple PAHs exposure with oxidative stress within the Korean adult population, using serum gamma glutamyltransferase (GGT) as an oxidative stress marker. Data from the Second Korean National Environmental Health Survey (2012–2014) were analyzed. For analysis, 5225 individuals were included. PAH exposure was assessed with four urinary PAH metabolites: 1-hydroxyphenanthrene, 1-hydroxypyrene, 2-hydroxyfluorene, and 2-naphthol. After adjusting for age, sex, body mass index, drinking, passive smoking, and current smoking (model 1), as well as the presence of diabetes and hepatobiliary diseases (model 2), complex samples general linear model regression analyses for each metabolite revealed a significant positive association between Ln(1-hydroxyphenanthrene) and Ln(GGT) (model 1: β = 0.040, p < 0.01 and model 2: β = 0.044, p < 0.05). For the complete dataset (n = 4378), a significant positive association was observed between mixture of four urinary PAH metabolites and serum GGT in both the quantile g-computation and the Bayesian kernel machine regression analysis. Our study provides evidence for the association between mixed PAH exposure and oxidative stress.

## Introduction

Reactive oxygen species (ROS) production beyond the antioxidant defense capability of body leads to oxidative stress^1^. Oxidative stress and associated systemic inflammation can influence the progression of diseases such as cardiovascular and neurodegenerative diseases^2,3^. Exposure to pollutants such as particulate matter (PM) and irritant gases has been linked to various adverse health effects. Oxidative stress triggered by these pollutants has been suggested as a plausible mechanism for these associations^4,5^.

Polycyclic aromatic hydrocarbons (PAHs) are pervasive pollutants that are associated with a range of negative health effects. PAHs are typically produced from incomplete combustion of organic carbon compounds^6^, with primary sources including vehicle and industrial emissions, wood and coal burning, and tobacco smoking^7^. PAHs can also be present in various food products due to factors such as environmental pollution and cooking processes, and therefore food can be a source of PAHs exposure^8^. Like other pollutants, PAHs are known to cause cancers in humans and animals^9,10^ and have been associated with other health conditions including cardiovascular diseases (CVDs), allergic diseases, and neurodegeneration^11–13^. PAHs-induced ROS is thought to be responsible for carcinogenic DNA damage^14–16^, and oxidative stress due to ROS production may contribute to the harmful health effects of PAH exposure.

Several studies have examined the relationship between PAH exposure and oxidative stress in humans. For example, in schoolchildren from China and South Korea, urinary 1-hydroxypyrene was positively associated with urinary malondialdehyde (MDA), an oxidative stress biomarker^17^. A dose–response relationship was observed between urinary metabolites of PAHs and the oxidative stress biomarker urinary 8-hydroxy-2'-deoxyguanosine (8-OHdG) in a Chinese population^18^. In another study, average oxidative stress biomarker levels (8-OHdG and MDA) were higher in workers highly exposed to PAHs than in those mildly exposed and the control group, although these differences were not significant^19^. Although many studies have suggested an association between PAH exposure and oxidative stress in human populations, these associations differ depending on the PAH metabolites evaluated in each study. Also, there is a dearth of studies assessing the overall impact of mixed PAH exposure on oxidative stress.

Gamma glutamyltransferase (GGT) is vitally involved in maintaining the homeostasis of intracellular glutathione, which exhibits intracellular antioxidant activity, facilitating the degradation of extracellular glutathione and promoting the synthesis of intracellular glutathione^20^. Serum GGT has been suggested as an oxidative stress biomarker of early phase within the normal range^21–25^, although it is primarily used as a marker for hepatobiliary diseases. This study evaluated the relationship of mixed PAH exposure with oxidative stress using serum GGT levels in the Korean adult population.

## Methods

### Study participants

This study analyzed the data derived from the Second Korean National Environmental Health Survey (KoNEHS), conducted by the National Institute of Environmental Research (NIER) between 2012 and 2014. KoNEHS, which has a cross-sectional nature, is a nationwide biomonitoring survey designed to identify the extent and source of exposure to environmental pollutants in Korean population^26^. The survey applied stratified two-stage sampling and included 6,478 subjects aged 19 and over from 400 districts, reflecting the population distribution. Data were gathered via personal interviews and biological sampling. A subset of the KoNEHS population was selected for analysis (n = 5225), comprising individuals with serum GGT levels lower than 100 U/L without missing values, and with no excessive alcohol use. Because high GGT levels may indicate a disease state regardless of oxidative stress, individuals with GGT levels above 100 U/L were excluded from the analysis. Additionally, individuals with excessive alcohol use were excluded because it can affect GGT independently of oxidative stress. Excessive alcohol use included binge drinking (n = 165) (about ≥ 4 drinks per occasion for women; ≥ 5 for men) and heavy drinking (n = 921) (about ≥ 8 drinks per week for women; ≥ 15 for men). A single drink was defined as approximately 14 g of alcohol per serving^27^.

### Serum GGT, urine PAH metabolites, and covariates

Serum GGT levels was employed as an oxidative stress marker. PAH exposure was assessed through urinary PAH metabolites: 1-hydroxyphenanthrene, 1-hydroxypyrene, 2-hydroxyfluorene, and 2-naphthol. Naphthol, hydroxyfluorene, hydroxyphenanthrene, and hydroxypyrene, which are urinary metabolites of low- to medium-molecular-weight PAHs including naphthalene, fluorene, phenanthrene and pyrene, constitute the majority of urinary PAH metabolites, and these urinary hydroxy-PAHs are commonly used as exposure biomarkers for PAHs^28,29^. Detailed information on the method of analysis has been described elsewhere^26,30–32^. In brief, levels of serum GGT and urinary PAH metabolites were based on individual blood and spot urine samples, respectively. Both samples were taken at the same time. Serum GGT levels were evaluated using colorimetry, and concentrations of urinary PAH metabolites were assessed with gas chromatography–mass spectrometry (GC–MS). The analytical range for serum GGT was 4.0–1,200 U/L. The limits of detection (LODs) for each metabolite were 0.047 µg/L for 1-hydroxyphenanthrene, 0.015 µg/L for 1-hydroxypyrene, 0.04 µg/L for 2-hydroxyfluorene, and 0.05 µg/L for 2-naphthol. Concentrations below the LOD for each metabolite were substituted by a value which is each metabolite's LOD divided by the square root of 2. Urinary PAH metabolite concentrations were adjusted by urine creatinine concentrations in the range of (0.3–3.0 g/L). Each urinary PAH metabolite was sorted into four groups by quartiles.

Variables included age, sex, body mass index (BMI), current drinking status, current smoking status, and the presence of passive smoking. BMI was divided into < 18 kg/m^2^ *vs.* 18 ≤ and < 25 kg/m^2^ *vs.* 25 ≤ kg/m^2^. Drinking status was divided as currently drinking vs. currently not drinking. Smoking status was grouped as currently smoking vs. currently not smoking (including ex- and non-smokers). Additionally, the presence or absence of diabetes or hepatobiliary diseases was also included as variables since these can affect the level of serum GGT^22,33^. The response rate for questionnaire items related to the current history of diseases such as diabetes and hepatobiliary disease was 46.2% (n = 2999).

### Statistical analyses

Stratum, cluster, and weight were incorporated into analyses because the KoNEHS has the stratified two-stage cluster sampling structure. Given that the distributions of serum GGT and urinary PAH metabolite concentrations were skewed, they were subjected to logarithmic transformation. The estimated geometric means (GMs) of serum GGT levels were compared across quartile groups of four urinary metabolites of PAHs in the total KoNEHS population (n = 6478) and subpopulation (n = 5225). The relationships between single urinary PAH metabolites and serum GGT levels were assessed for the subpopulation using complex samples general linear model (CSGLM) regression analyses. Age, sex, BMI, drinking, and smoking status were adjusted (model 1), and the presence of diabetes or hepatobiliary diseases was also taken into account (model 2). Complex samples statistical analyses were performed using SPSS v25 for Windows (IBM, Armonk, NY, USA).

Quantile g-computation (qg-computation) and Bayesian kernel machine regression (BKMR) were used to assess the mixed effect of PAH exposures on serum GGT. Qg-computation evaluates the effect of a simultaneous increase in one quantile of every exposure on the outcome by estimating the parameters of a marginal structural model^34^. Also, qg-computation evaluates for both positive and negative effects of exposures on the outcome^34^. BKMR is a nonparametric method that allows the application of kernel function to assess the joint effects of exposures, considering nonlinear relationships and/or potential interactions^35^. For the complete dataset with no missing values for four urinary PAH metabolites (n = 4378), qg-computation and BKMR were conducted using the bkmr v0.2.2/ bkmrhat v1.1.3 packages and the *qgcomp* v2.10.1 package in R v4.3.1 (R Development Core Team, Vienna, Austria). Both models were adjusted for age, sex, BMI, current smoking, and current drinking. In the qg-computation model, 10-quantiles were applied for the exposure variables. The BKMR model was fitted using Markov chain Monte Carlo (MCMC) with 80,000 iterations which included 40,000 burn-in iterations. In the BKMR analysis, the default setting of the bkmr and bkmrhat packages was used, and posterior inclusion probabilities (PIPs) were computed to show the importance of each PAH metabolite in the mixture. Statistical significance was evaluated at *p* < 0.05.

### Ethics approval and consent to participate

This study using the 2nd KoNEHS data received approval from the Institutional Review Board of Inje University Haeundae Paik Hospital (No. 2022-11-013). The KoNEHS was approved by the Institutional Review Board of NIER and informed consent was obtained from all participants. All methods used in this study were performed in accordance with relevant guidelines and regulations.

## Results

Table 1 outlines the general characteristics of subpopulation (n = 5225) and total the KoNEHS population (n = 6478). The estimated percentage of subjects with values exceeding 100 U/L or missing values for serum GGT was 5.1% (n = 332) among total population. Subjects with excessive alcohol use constituted an estimated 20.5% of the total population (n = 1086). The distributions of each PAH metabolite for the entire population of the KoNEHS are shown in Supplementary Table S1.

**Table 1:** General characteristics of study population.

Variable	Subpopulation^a^ (n = 5225)	Total population (n = 6478)
	Estimated mean (SE) or unweighted n (estimated %)	Estimated mean (SE) or unweighted n (estimated %)
Age (years)	47.6 (0.4)	46.3 (0.4)
19–35	797 (25.8%)	1109 (28.3%)
36–50	1418 (31.3%)	1826 (32.3%)
51–65	1787 (25.9%)	2179 (24.9%)
65 <	1223 (17.0%)	1364 (14.5%)
Sex		
Male	1869 (42.1%)	2774 (49.2%)
Female	3356 (57.9%)	3704 (50.8%)
Current smoking		
No	4579 (85.1%)	5317 (78.5%)
Yes	646 (14.9%)	1161 (21.5%)
Passive smoking		
No	4150 (77.0%)	4937 (73.3%)
Yes	1075 (23.0%)	1541 (26.7%)
Current drinking		
No	2594 (44.6%)	2654 (35.3%)
Yes	2631 (55.4%)	3824 (64.7%)
BMI (kg/m^2^)		
< 18	68 (1.5%)	82 (0.2%)
18 ≤—< 25	3226 (64.3%)	3902 (61.6%)
25 ≤	1931 (34.3%)	2494 (36.9%)
Diabetes^b^		
No	2077 (82.8%)	2451 (83.2%)
Yes	457 (17.2%)	548 (16.8%)
Hepatobiliary diseases^b^		
No	2502 (98.9%)	2952 (98.4%)
Yes	32 (1.1%)	47 (1.6%)
Serum GGT (U/L)		
≤ 100	–	6146 (94.9%)
> 100^c^	–	332 (5.1%)
Excessive alcohol use		
No	–	5392 (79.5%)
Yes	–	1086 (20.5%)

^a^Subpopulation excluding subjects with serum GGT above 100 U/L or excessive alcohol use.

^b^The response rate for the questionnaire items related to current history of diseases was 46.2% (n = 2999).

^c^This category includes subjects who have missing values for serum GGT (n = 22).

Table 2 displays the estimated GMs of serum GGT by quartile groups of urinary PAH metabolites for the total population and subpopulation. The GMs of the fourth quartiles of each PAH metabolite significantly differed from those of the first quartiles in both the total population and subpopulation (*p* < 0.01). The estimated GMs of serum GGT appeared to increase significantly with the progression of quartiles in all PAH metabolites (*p* for trend < 0.01).

**Table 2:** Estimated geometric mean of serum GGT by quartile groups of urinary PAH metabolites.

Urinary metabolites	Subpopulation^a^ (n = 5225)		*p* for trend	Total KoNEHS population (n = 6478)		*p* for trend
	GM^b^	95% CI		GM^b^	95% CI	
1-Hydroxypyrene			< 0.01			< 0.01
≤ 25th percentile	19.8	18.8–20.8		22.1	21.0–23.3	
25 <—≤ 50th percentile	19.9	19.0–20.8		22.6	21.6–23.7	
50 <—≤ 75th percentile	20.4	19.5–21.4		24.5	23.1–26.0*	
> 75th percentile	22.2	21.2–23.2*		28.5	27.0–30.1*	
2-Naphthol			< 0.01			< 0.01
≤ 25th percentile	19.2	18.3–20.2		21.8	20.6–23.1	
25 <—≤ 50th percentile	19.6	18.7–20.4		21.9	21.0–23.0	
50 <—≤ 75th percentile	20.2	19.3–21.2		24.2	23.0–25.5*	
> 75th percentile	23.7	22.5–25.0*		30.2	28.6–31.8*	
1-Hydroxyphenanthrene			< 0.01			< 0.01
≤ 25th percentile	19.8	18.9–20.8		22.0	21.0–23.1	
25 <—≤ 50th percentile	19.8	18.9–20.8		22.9	21.7–24.2	
50 <—≤ 75th percentile	20.7	19.7–21.7		25.2	23.8–26.7*	
> 75th percentile	21.8	20.9–22.8*		27.5	26.1–29.0*	
2-Hydroxyfluorene			< 0.01			< 0.01
≤ 25th percentile	19.1	18.2–20.0		21.4	20.3–22.5	
25 <—≤ 50th percentile	19.2	18.4–20.1		21.0	20.0–22.2	
50 <—≤ 75th percentile	19.6	18.8–20.4		22.7	21.6–23.8	
> 75th percentile	26.2	24.8–27.6*		34.2	32.3–36.2*	

*GGT* gamma glutamyltransferase, *PAH* polycyclic aromatic hydrocarbon, *KoNEHS* Korean National Environmental Health Survey, *GM* geometric mean, *CI* confidence interval.

^a^Subpopulation excluding subjects with serum GGT above 100 U/L or excessive alcohol use.

^b^Estimated geometric mean and 95% confidence interval from complex samples general linear model regression.

*Significantly different compared to first quartile (*p* < 0.01).

The associations of serum GGT levels with the concentration of each urinary PAH metabolite in the subpopulation, as assessed using CSGLM regression, are shown in Table 3. 1-Hydroxyphenanthrene was significantly positively associated with serum GGT in both model 1 (β = 0.040, *p* < 0.01) and model 2 (β = 0.044, *p* < 0.05). 2-Hydroxyfluorene showed a significant association with serum GGT only in model 1 (β = 0.034, *p* < 0.05). We found no significant associations with serum GGT among the other metabolites.

**Table 3:** Association between serum GGT levels and urinary PAH metabolites in subpopulation^a^ (n = 5225).

Urinary metabolites	CSGLM	β	SE	*p*-value
1-Hydroxypyrene				
Model 1		0.012	0.014	0.401
Model 2		0.026	0.021	0.204
2-Naphthol				
Model 1		0.014	0.010	0.162
Model 2		0.018	0.012	0.133
1-Hydroxyphenanthrene				
Model 1		0.040	0.014	0.006
Model 2		0.044	0.020	0.027
2-Hydroxyfluorene				
Model 1		0.033	0.015	0.028
Model 2		0.017	0.018	0.364

*GGT* gamma glutamyltransferase, *PAH* polycyclic aromatic hydrocarbon, *CSGLM* complex samples general linear model, *SE* standard error, *BMI* body mass index.

^a^Subpopulation excluding subjects with serum GGT above 100 U/L or excessive alcohol use.

Model 1: Ln(GGT) = age + sex + BMI + current smoking + passive smoking + current drinking + Ln(each urinary PAH metabolite).

Model 2: Ln(GGT) = age + sex + BMI + current smoking + passive smoking + current drinking + diabetes + hepatobiliary diseases + Ln(each urinary PAH metabolite).

**Figure 1:**
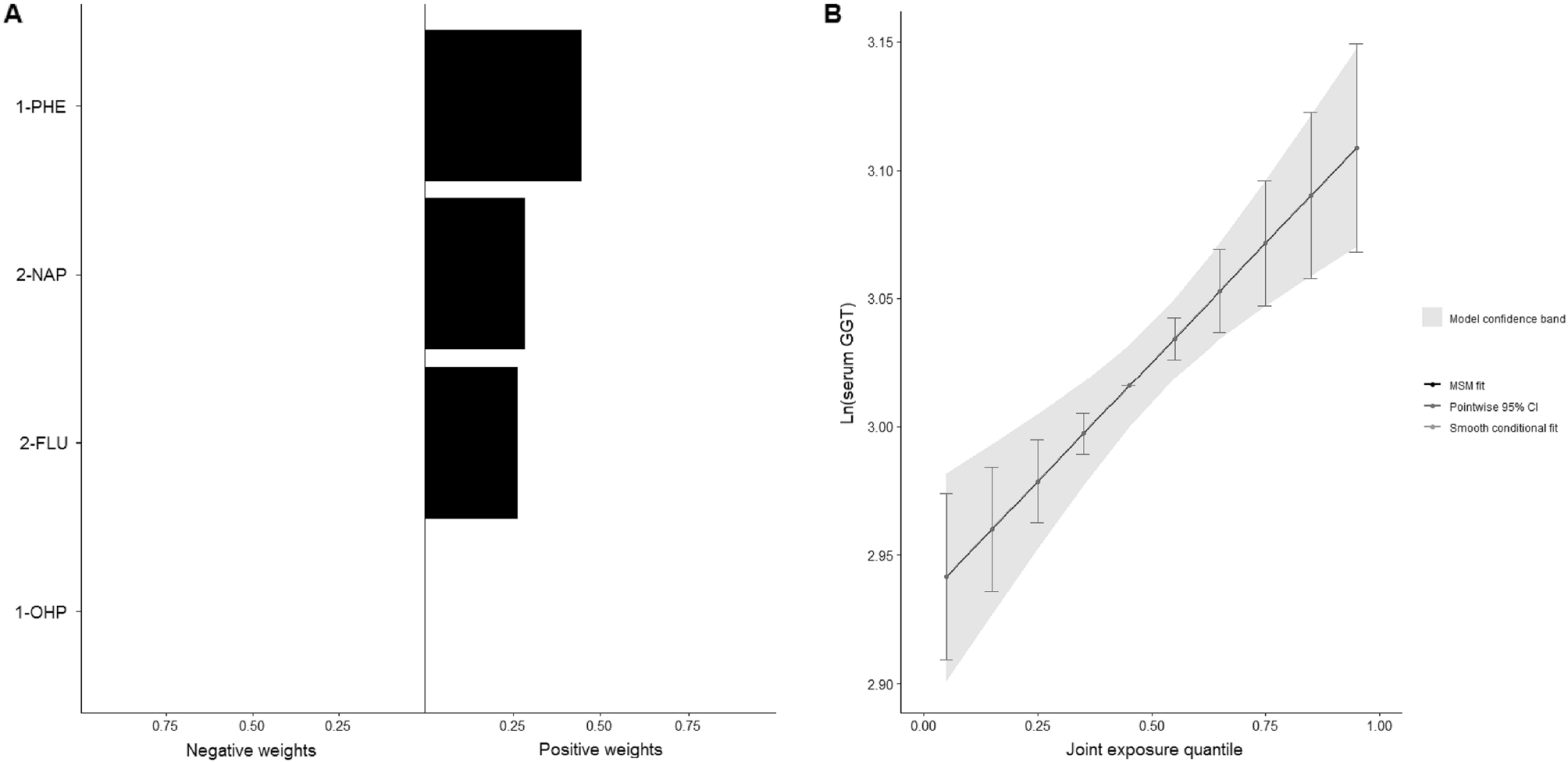
Associations of PAH metabolites with serum GGT from quantile g-computation in the complete dataset (n = 4378). (**A**) Weights of each PAH metabolite, and (**B**) the joint intervention levels of PAH metabolite mixtures on Ln(serum GGT). Quantile g-computation regression model analysis with complete case dataset (n = 4378) among subpopulation; adjusted for age, sex, BMI, current smoking, and current drinking. *PAH* polycyclic aromatic hydrocarbon, *GGT* gamma glutamyltransferase, *1-PHE* 1-hydroxyphenanthrene, *2-NAP* 2-naphthol, *2-FLU* 2-hydroxyfluorene, *1-OHP* 1-hydroxypyrene.

Table 4 presents the mixture effect of the four PAH metabolites on serum GGT, as determined by the quantile g-computation regression. The mixed four urinary metabolites of PAHs showed a significant positive association with serum GGT levels (mixture β = 0.0186, *p* < 0.001). All PAH metabolites exhibited positive effects on serum GGT (Fig. 1A), with 1-hydroxyphenanthrene carrying the largest weight (1-hydroxyphenanthrene: 0.3772; 2-naphthol: 0.3087; 2-hydroxyfluorene: 0.2457; 1-hydroxypyrene: 0.0685). The joint intervention levels of the PAH metabolite mixtures on serum GGT are illustrated in Fig. 1B.

**Table 4:** The overall effect of the mixture of four PAH metabolites on serum GGT in the complete dataset (n = 4378)^a^.

	Mixture slope	SE	*p*-value	Positive^c^	Negative^d^
Serum GGT^b^	0.0186	0.0042	< 0.001	0.0186	0

^a^Quantile g-computation model regression analysis with complete case dataset (n = 4378) among subpopulation; adjusted for age, sex, BMI, current smoking, and current drinking.

^b^Ln(serum GGT).

^c^Sum of positive coefficients.

^d^Sum of negative coefficients.

*PAH* polycyclic aromatic hydrocarbon, *GGT* gamma glutamyltransferase, *SE* standard error, *BMI* body mass index.

**Figure 2:**
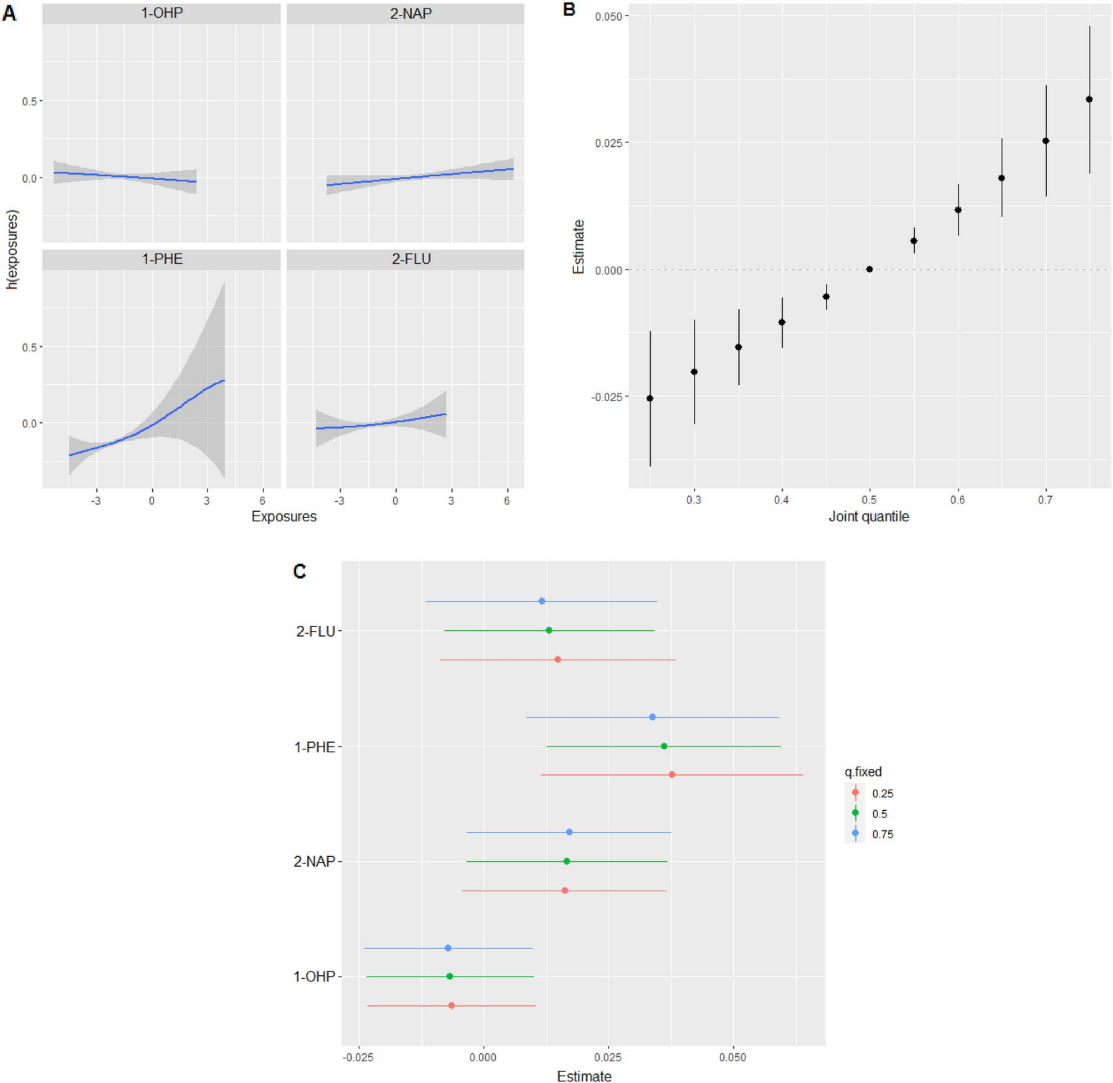
Associations of PAH metabolites with serum GGT from Bayesian kernel machine regression in the complete dataset (n = 4378). (**A**) Univariate exposure–response function *h*(exposure) of each PAH metabolite, where all other PAH metabolites were fixed at their 50th percentiles. (**B**) Joint effect of mixed PAH metabolites on serum GGT, calculated by comparing the outcome when all PAH metabolites were at a specific percentile from the 25th to 75th percentile with when all metabolites were at the 50th percentile. C. Individual effects of each PAH metabolite on serum GGT outcome, representing changes of outcome in each PAH metabolite from its 75th to its 25th percentile, where other PAH metabolites were fixed at the 25th, 50th, and 75th percentiles. The dots represent the estimate, and the lines depict the 95% credible intervals. All PAH metabolites were log-transformed. The model was adjusted for age, sex, BMI, current smoking, and current drinking. *1-OHP* 1-hydroxypyrene, *2-NAP* 2-naphthol, *1-PHE* 1-hydroxyphenanthrene, *2-FLU* 2-hydroxyfluorene, *PAH* polycyclic aromatic hydrocarbon, *GGT* gamma glutamyltransferase, *BMI* body mass index.

Table 5 presents the posterior inclusion probabilities (PIPs) for each PAH metabolite in the BKMR model. Among the metabolites, 1-hydroxyphenanthrene was most strongly associated with serum GGT (PIP = 0.8402). Figure 2 shows associations between PAH metabolites and serum GGT using the BKMR model. Figure 2A illustrates the univariate associations between each PAH metabolite and serum GGT when fixing others to the median, showing that urinary 1-hydroxyphenanthrene has a significant positive association. Figure 2B shows the overall effect of the mixture of PAH metabolites on serum GGT and there was a strong positive association between the mixture of PAH metabolites and serum GGT. In the evaluation of individual effects (Fig. 2C), higher level of 1-hydroxyphenanthrene was significantly associated with higher serum GGT outcome.

**Table 5:** Posterior inclusion probability for each PAH metabolite on serum GGT in the complete dataset (n = 4378).

Urinary metabolites	PIPs
1-Hydroxypyrene	0.0035
2-Naphthol	0.0029
1-Hydroxyphenanthrene	0.8402
2-Hydroxyfluorene	0.1655

*PIP* posterior inclusion probability, *PAH* polycyclic aromatic hydrocarbon, *GGT* gamma glutamyltransferase.

## Discussion

This study examined the relationship of single and mixed PAH exposure with serum GGT. Among the individual PAHs, urinary 1-hydroxyphenanthrene showed a significant positive association with serum GGT. For combined PAH exposure, the mixture of four urinary PAH metabolites was positively associated with serum GGT levels in both quantile g-computation and BKMR analysis. Within the normal range, serum GGT has been suggested as an oxidative stress biomarker due to the intracellular antioxidant activity of GGT^24^. Our findings imply that PAH exposure is positively correlated with oxidative stress.

Several studies have evaluated the association between PAH exposure and oxidative stress. For example, there was a significant positive correlation between urinary 1-hydroxypyrene and 8-OHdG in coke-oven workers^36^, and urinary 1-hydroxypyrene was significantly associated with 8-OHdG in kitchen staff and asphalt workers^37,38^. Urinary 1-hydroxypyrene was associated with urinary MDA in schoolchildren^17^, and a dose–response relationship was observed between urinary PAH metabolites and 8-OHdG in the general population of China^18^. A German population study found significantly positive relationships of urinary hydroxy-PAHs with biomarkers of oxidative stress, including MDA, 8-OHdG, and F2α-isoprostanes^39^. Similarly, in the Chinese population, urinary hydroxy-PAHs had positive associations with urinary MDA^40,41^. Also, in terms of oxidative stress, the relationships between PAH metabolites and serum GGT have been evaluated in several studies, with recent studies indicating a significant association between urinary PAH metabolites and serum GGT in the US adult^42^ and adolescent^43^ populations.

Our study revealed that the mixture of four urinary PAH metabolites had significant positive associations with serum GGT levels after adjusting for covariates, with 1-hydroxyphenanthrene having the most substantial impact. These results align with those of several prior studies. The increase in urinary 1-hydroxyphenanthrene was shown to be the most substantial estimated percentage change in urinary 8-OHdG among various PAH metabolites^18^. Another study indicated that a 100% increase in the sum of urinary hydroxyphenanthrenes was associated with a 22.4% increase in MDA, which represented the largest percent change compared to other PAH metabolites among healthy subjects^41^. Among PAH metabolites, 1-hydroxyphenanthrene was found to be the most strongly associated with serum GGT levels, followed by 2- and 3-hydroxyphenanthrene^42^.

However, associations with oxidative stress have yielded different results depending on the PAH metabolites evaluated, and few studies have considered combined exposure to multiple PAHs. A pilot study of the Chinese population evaluated the joint effect of hydroxy-PAHs on 8-OHdG, as well as oxygen radical antioxidant capacity (ORAC) and hydroxyl radical antioxidant capacity (HORAC), which are employed as indicators of antioxidant capacity, using BKMR^44^. The BKMR models indicated positive relationships between the eight hydroxy-PAHs and urinary 8-OHdG, as well as plasma ORAC and HORAC activity. Moreover, urinary 2- plus 3-hydroxyphenanthrene contributed significantly to the increase in urinary 8-OHdG levels among a mixture of urinary PAH metabolites^44^. Another study evaluated the association between mixed PAH exposure and serum GGT in relation to liver function using weighted quantile sum regression in the US adolescent population^45^. However, unlike our study, the study did not detect a significant association between serum GGT and either individual or mixed urinary PAH metabolites. This discrepancy could be attributed to variations in the age groups of the study populations or the levels or durations of PAH exposure between studies. Antioxidant defenses may also vary among age groups^46,47^.

Several mechanisms have been proposed for the association of PAH exposure with oxidative stress. PAHs are metabolized through cytochrome P450 enzymes (CYPs) such as CYP1A1/2 and CYP1B1, and then become reactive intermediates that can covalently bind with DNA, causing carcinogenicity, and can establish redox cycles, leading to ROS generation^16^. PAH exposure may also be involved in regulating ROS-generating enzymes such as CYPs, through the aryl hydrocarbon receptor signaling pathway, leading to oxidative stress^48^.

Beyond the carcinogenic properties of PAHs, numerous studies have demonstrated associations of PAH exposure with other diseases, including CVDs^49^, allergic diseases^50^, diabetes^51^, and neurodegenerative diseases^13^. Although the underlying mechanisms are not well understood, oxidative stress induced by PAH exposure could be an underlying mechanism for these associations. Oxidative stress can cause chronic inflammation in the human body, which affects the progression of various diseases^52^. Oxidative stress related-inflammation has been proposed as a mechanism for endothelial dysfunction and arterial damage, which lead to CVDs^53^. Serum GGT has also been proposed as a potential marker for CVDs^22,54^, and this is thought to be related to its role as a marker of oxidative stress. Chronic oxidative stress can affect the regulation of neuroinflammatory response and cause neuroinflammation, leading to neurodegenerative diseases such as Parkinson’s and Alzheimer’s diseases^55^. ROS are also linked to the induction of allergic inflammation^56^.

This is the first study to evaluate the relationship between PAH exposure and oxidative stress from the perspective of mixed PAHs in Korean adults. We employed two independent analytical methods to evaluate the mixture effect, and found a positive association between mixed PAH exposure and oxidative stress. Previously, there have been few studies assessing association between mixed PAH exposure and oxidative stress. Nevertheless, our study has some limitations. Firstly, it is limited in evaluating causal relationships due to the cross-sectional nature of KoNEHS. Secondly, only serum GGT was included as an oxidative stress marker in this study because other oxidative stress markers such as MDA and 8-OHdG were not investigated in the second KoNEHS. Thirdly, due to the short half-lives of PAH metabolites, urinary levels of PAH metabolites in spot urine may not fully reflect the actual history of PAH exposure. However, since this is a study using national survey data, it can be helpful in understanding overall trends in the population.

## Conclusion

This study revealed a significant association between mixed PAH exposure and oxidative stress, providing an evidence for the overall effect of complex PAH exposure on oxidative stress response. Further studies are required to clarify this association.

### Supplementary Information

Supplementary Table S1.

## Data Availability

This study used data from the Second Korean National Environmental Health Survey (2012–2014), which was conducted by National Institute of Environmental Research (NIER), Republic of Korea. The data will be available on request to the NIER.

## Abbreviations

PAHs: Polycyclic aromatic hydrocarbons
GGT: Gamma glutamyltransferase
CSGLM: Complex samples general linear model
ROS: Reactive oxygen species
PM: Particulate matter
CVDs: Cardiovascular diseases
8-OHdG: 8-Hydroxy-2’-deoxyguanosine
MDA: Malondialdehyde
KoNEHS: Korean National Environmental Health Survey
NIER: National Institute of Environmental Research
GC–MS: Gas chromatography-mass spectrometry
LOD: Limits of detection
BMI: Body mass index
GM: Geometric mean
qg-computation: Quantile g-computation
MCMC: Markov chain Monte Carlo
PIP: Posterior inclusion probability
IRB: Institutional review board
ORAC: Oxygen radical antioxidant capacity
HORAC: Hydroxyl radical antioxidant capacity
BKMR: Bayesian kernel machine regression
WQS: Weighted quantile sum
CYPs: Cytochrome P450 enzymes
SE: Standard error
CI: Confidence interval
1-PHE: 1-Hydroxyphenanthrene
2-NAP: 2-Naphthol
2-FLU: 2-Hydroxyfluorene
1-OHP: 1-Hydroxypyrene
