# Supplementary material for: Association of mixed polycyclic aromatic hydrocarbons exposure with oxidative stress in Korean adults
Source: Sci Rep. 2024 Mar 29;14:7511. doi: 10.1038/s41598-024-58263-9 (PMC10980696; doi:10.1038/s41598-024-58263-9)
Supplement: Supplementary file 1 — Supplementary Table S1. [file 41598_2024_58263_MOESM1_ESM.pdf]

Supplementary Table S1. Distribution of four urinary PAH metabolites in the Second Korean National Environmental Health Survey.

Urinary metabolites	LOD (μg/L)	Percentage below LOD (%)	Estimated percentile (μg/g Cr)				
			5th	25th	50th	75th	95th
1-Hydroxypyrene	0.015	2.8	0.0601	0.1280	0.2009	0.3121	0.6517
2-Naphthol	0.050	1.0	0.5429	1.3281	2.8019	7.4000	20.6370
1-Hydroxyphenanthrene	0.047	21.0	0.0410	0.0777	0.1214	0.1934	0.3809
2-Hydroxyfluorene	0.040	7.3	0.0923	0.1883	0.3097	0.7073	2.0524

LOD; limit of detection, PAH; polycyclic aromatic hydrocarbon.
